# Sustainable and Biomimetic Methodology for Extraction of High-Value-Added Compounds in Almond Hulls

**DOI:** 10.3390/molecules29133034

**Published:** 2024-06-26

**Authors:** Gabriela Cremasco, Adam T. Sutton, Cristiano S. Funari, Dario R. Arrua, Kelly J. Dussan, Emily F. Hilder, Vanderlan S. Bolzani, Daniel Rinaldo

**Affiliations:** 1 Institute of Chemistry, São Paulo State University (UNESP), R. Prof. Francisco Degni 55, Araraquara 14800-900, SP, Brazil; gabriela.cremasco@unesp.br (G.C.); kelly.medina@unesp.br (K.J.D.); vanderlan.bolzani@unesp.br (V.S.B.); 2 Future Industries Institute, Mawson Lakes Campus, University of South Australia, Adelaide, SA 5095, Australia; adam.sutton@mymail.unisa.edu.au (A.T.S.); emily.hilder@unisa.edu.au (E.F.H.); 3 Green Biotech Network, School of Agricultural Sciences, São Paulo State University (UNESP), Av. Universitária, nº 3780-Altos do Paraíso, Botucatu 18610-034, SP, Brazil; cristiano.funari@unesp.br; 4 Green Biotech Network, School of Sciences, São Paulo State University (UNESP), Av. Eng. Luiz Edmundo Carrijo Coube 14-01, Bauru 17033-360, SP, Brazil

**Keywords:** *Prunus dulcis*, design of experiments, green chemistry, NADESs, Microwave-Assisted Extraction

## Abstract

Almond trees are the most cultivated nut tree in the world. The production of almonds generates large amounts of by-products, much of which goes unused. Herein, this study aimed to develop a green chemistry approach to identify and extract potentially valuable compounds from almond by-products. Initially, a screening was performed with 10 different Natural Deep Eutectic Solvents (NADESs). The mixture lactic acid/glycerol, with a molar ratio 1:1 (1:50 plant material to NADES (*w*/*v*) with 20% *v*/*v* of water) was identified as the best extraction solvent for catechin, caffeoylquinic acid, and condensed tannins in almond hulls. Subsequently, a method was optimized by a Design of Experiment (DoE) protocol using a miniaturized extraction technique, Microwave-Assisted Extraction (MAE), in conjunction with the chosen NADESs. The optimal conditions were found to be 70 °C with 15 min irradiation time. The optimal extraction conditions determined by the DoE were confirmed experimentally and compared to methods already established in the literature. With these conditions, the extraction of metabolites was 2.4 times higher, according to the increase in total peak area, than the established literature methods used. Additionally, by applying the multiparameter Analytical Greenness Metric (AGREE) and Green Analytical Process Index (GAPI) metrics, it was possible to conclude that the developed method was greener than the established literature methods as it includes various principles of green analytical chemistry.

## 1. Introduction

Almond trees (*Prunus dulcis*) are the most cultivated tree nut in the world, with a production of more than 1.5 metric tons of whole nuts in the 2022/2023 season [1]. The largest producer is the United States, followed by Australia and Spain, with an increasing production trend [1].

The almond fruit is divided into a greenish-colored casing called the hull, a hard and porous intermediate layer called the shell, a brown skin that surrounds the nut, protecting it and preventing oxidation and contamination, as well as the nut itself. Combined, the hull, shell, and skin represent approximately 85% of the mass of the almond fruit, the hull being the most abundant part [2,3,4]. Finding high-value-added applications for all parts of the almond fruit would provide value in the almond production chain. Additionally, finding useful applications would be in line with the goals of the United Nations (UN) 2030 agenda, more specifically with goals 9 and 12 to build resilient infrastructure, promote inclusive and sustainable industrialization, and foster innovation, as well as to ensure sustainable consumption and production patterns [5]. Currently, almond components other than the nut are typically used as animal feed or in energy production [3].

One promising option to add value to the almond production chain is the sustainable exploration of almond by-products as a potential long-term source of bioactive natural products [3]. The shell has a high xylan content—which can be fractionated into cellulose, pentosans, and lignins—which has been studied for water resistance, demonstrating that the addition of almond shell particles to wood increased the waterproofness of the wooden panels studied [6]. Lignocellulosic biomass from almond shells has also been suggested as a renewable organic carbon source as its combustion, compared to petroleum derivatives, emits fewer hydrocarbons and monoxides [4]. Almond hulls are often overlooked as a by-product in almond production. However, they have been studied extensively for their phenolic constituents and antioxidant properties. Sang et al. [7] isolated several compounds from almond hulls, including protocatechuic acid and catechin, both known for their strong antioxidant capabilities, with their 2,2-diphenyl-1-picrylhydrazyl (DPPH) Radical Scavenging Capacity being above 90%. Barreira et al. evaluated almond hulls for their antioxidant properties, obtaining a DPPH Radical Scavenging Capacity above 80% at a concentration of 0.5 mg/mL [8]. The development of highly efficient and sustainable processes for identifying and extracting these types of compounds is needed to accelerate efforts in the area.

The U.S. Food and Drug Administration (FDA) recommends that all parameters that may interfere in a process be studied [9]. To assess multiple parameters in a systematic, simultaneous, and unbiased approach, a Design of Experiments (DoE) protocol can be used. Multivariate optimizations not only allow for detection of synergy between traditional extraction parameters, but they also allow for a greener optimization as they avoid the additional experiments that would be needed to optimize parameters one at a time [10]. With a focus on green extraction processes, low-energy consuming, fast, and efficient extraction techniques are needed for studying almonds. One example is Microwave-Assisted Extraction (MAE) [11]. Moreover, the selection of the extraction solvent is important since it has the greatest impact on extraction efficiency and the greenness as such solvents create potentially hazardous waste [10,12,13]. Finding extraction solvents that lead to efficient extractions, while maintaining green processes, is not a trivial task as most extraction solvents are derived from petroleum. One alternative is offered by Natural Deep Eutectic Solvents (NADESs) [14,15].

NADESs are eutectic mixtures of two or more natural compounds, generally from the primary metabolism of plants, which can remain liquid at room temperature [16]. The advantages of NADESs can include their easy preparation, biodegradability, renewability, biocompatibility, and ability to extract secondary metabolites [12]. In 2018, the company Naturex^®^ (U. S. Patent No. 0055904, 2018) filed a patent reporting the use of a eutectic solvent composed of natural molecules for the efficient extraction of plant materials, demonstrating the practical and commercial potential of NADESs [17]. Additionally, Naturex^®^ has a line of NADES-based extracts for the cosmetic industry known as Eutectys™ [18]. NADESs can be classified into five categories, depending on the nature of the compounds used in their synthesis: (i) ionic liquid type, composed of an acid and a base; (ii) neutral type, formed by sugars or a combination of sugars and polyalcohols; (iii) neutral-acid type, synthesized from a sugar or polyalcohol mixed with an organic acid; (iv) neutral-basic type, with a sugar or polyalcohol mixed with an organic base; and v) amino acid type, which contains amino acids combined with sugars or organic acids [19]. This classification reflects the diversity of NADESs available, making it possible to optimize extractions based on the matrix and bioactive compounds desired by selecting different NADESs. Continuous advances in knowledge and understanding of the characteristics and applications of NADESs have driven the exploration of these solvents as promising alternatives for various industrial purposes [15,19]. An additional advantage of using NADESs as an extraction solvent is that those made from food components (those that are formulated with natural ingredients and safe for human consumption) can be used for the delivery of bioactive compounds directly without removal of the solvent [17].

NADESs have great potential as alternative solvents, especially in the removal of bioactive compounds from plants. They improve the degradation capacity, solubility, stability, bioactivity, and bioavailability of these compounds [20]. Gomez-Urios et al. [21] evidenced that orange peel extracts prepared with NADESs contained more bioactive compounds, such as catechin and caffeic acid, compared to ethanol, avoiding a high layer between these bioactive compounds and the eutectic solvents. Another study also highlighted the use of NADESs to improve thermal and storage stability in addition to the antimicrobial activity of catechins. The interaction between NADESs and catechins forms hydrogen bonds, explaining the high stability of catechins in these solvents [22].

One of the disadvantages of NADESs is their non-volatile nature, due to high viscosity and high boiling points, making them difficult to remove after extraction [23]. Despite this difficulty, studies show great potential to produce ready-to-use extracts without the need to remove the eutectic solvent, increasing the bioavailability of phenolic compounds from plant matrices. [23]

Herein, this work proposes an innovative green method for the extraction of by-products from almond hulls. The research strategy adopted was based on the principles of sustainable analytical chemistry, using a multivariate experimental optimization (called central composite design, CCD), reducing the overall waste and resources needed for the method development. MAE using NADESs as extracting solvents (NADES–MAE) was evaluated as an improved extraction method for identifying and collecting value-added compounds from almond by-products. The feasibility and effectiveness of this method was thoroughly evaluated, and directly compared to established methods from the literature, both in terms of analytical extraction performance and greenness, using two complementary multiparametric green metrics.

## 2. Results and Discussion

### 2.1. Identification of Compounds Present in Almond Hull Extracts

The extract obtained from the Pinelo et al. [24] extraction method (methanol and water dynamic maceration) was used for compound identification, as among the reference methods, it had the most peaks in the chromatogram.

**Figure 1:**
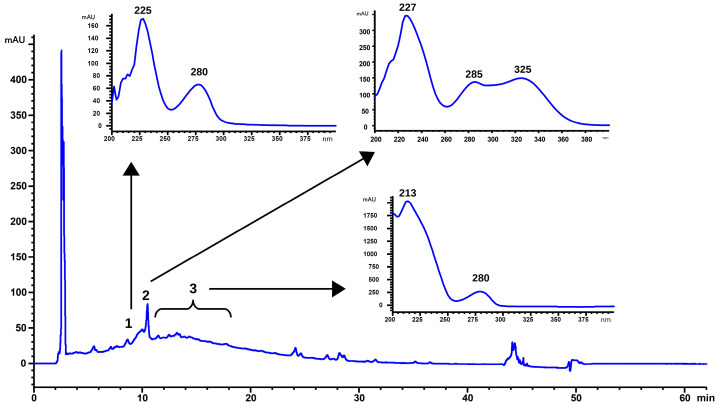
Representative chromatogram of almond hull hydromethanolic acid extract (extraction method reproduced from Pinelo el al. [24]), monitored at 254 nm and UV spectrum of the main phenolic compounds: (1) catechin derivative; (2) caffeic acid derivative; and (3) condensed tannins (HPLC conditions described in Section 3.5).

Initially, HPLC–DAD was used to identify the classes of metabolites present in the extract. Additional peak identification was carried out by comparing the UV spectra and retention times of peaks present in the chromatograms with reference standards. The classes identified were as follows: (1) catechin derivative, (2) caffeic acid derivative, and (3) condensed tannins (Figure 1).

LC–HRMS analysis was performed to further identify and confirm compounds extracted. In the negative mode, extracted ion chromatograms, a deprotonated molecular series corresponding to the homologous series of type B proanthocyanidins with a degree of polymerization of one to five linked units of catechin, was observed (([M − H]^−^) values shown in Table 1 (Figures S1–S4)). The ion *m*/*z* 289 (catechin monomer) is present with greater relative intensity (retention time 9 min). As the number of polymerization units increases, there is an increase in retention time, which suggests that tannins are present.

Therefore, peak 1 (9 min, Figure 1) may belong to a catechin/epicatechin monomer. In peak 2, whose UV spectrum represents a derivative of caffeic acid, the presence of the ion *m*/*z* 353 ([M − H]^−^) was observed, which suggests the presence of caffeoylquinic acid. The absorptions from 11 to 30 min represent tannins of two to five units, similar to the UV spectrum of catechin/epicatechin monomer.

### 2.2. Screening of NADESs as Extractive Solvents by MAE

The extraction efficiency for the target compounds was the most important parameter for comparing the NADESs used. The extraction efficiency was measured based on the total peak area in the chromatograms where the larger the peak area, the greater the quantity of compounds extracted. It is important to highlight that in calculating the peak areas, the areas referring to the compounds that constitute the NADESs were disregarded, by subtracting a pure NADES (blank) from the sum of the peak areas of the extract chromatograms.

**Figure 2:**
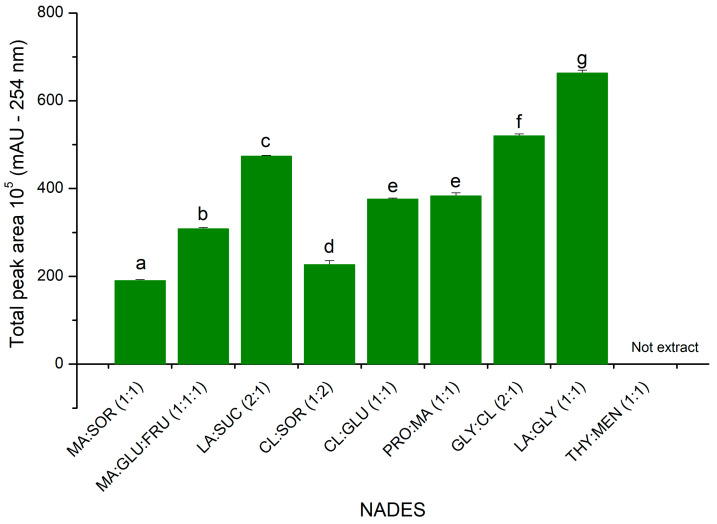
Comparison of total peak areas obtained from NADES extractions (n = 3). Different letters represent a statistically significant difference: *p* < 0.0005.

The NADESs that showed the best extraction yield were N8 (GLY:CL 2:1) and N9 (LA:GLY 1:1) (Figure 2, Table S1). This result is likely related to these two NADESs having a lower viscosity than the other NADESs, which improves mass transports [25,26]. Although N10 (THY:MEN 1:1, Table 2) is also not very viscous, it did not extract the phenolic metabolites present in almond hulls, which may be related to its hydrophobic nature.

NADES 9 (GLY:LA) contains an acidic component which reduces the pH of the extraction mixture, which can have advantages for several applications. The acidic pH of NADES 9 acts similarly to an acid catalyzed extraction process, enabling the increased extraction of bioactive compounds [27].

For NADESs, the densities and viscosities of eutectic mixtures are adjustable parameters through the addition of water. Reducing the viscosity makes the NADESs easier to handle, which is essential in various industrial processes [28,29]. However, it is important to note that the addition of water in quantities greater than 49% by mass of NADES will result in the breaking of the hydrogen bonds present. This, in turn, will lead to dissolution of the supramolecular structure of the eutectic mixture, as observed in previous studies [30,31]. Furthermore, Wei et al. [28] also observed that the addition of 20% water to a NADES resulted in a significant improvement in the extraction of metabolites of varying polarities, not only the water-soluble compounds. This suggests that this amount of water can reduce the viscosity of NADESs and improve the extraction yield, while preserving the supramolecular structure of these eutectic solvents, as also discussed by Fraige et al. [32], Dai et al. [30], and Bonacci et al. [33]. For this reason, in this study, it was decided to add 20% of water in relation to the total mass of hydrophilic NADES evaluated (all NADESs except N10). Overall, since N9 based on LA and GLY had the highest extraction yield, including phenolic compounds, it was used for further optimization of the extraction of almond hulls using MAE.

### 2.3. Optimization of Microwave-Assisted Extraction (MAE) by Multivariate Experimental Design

A 2^3^ complete factorial design with repetition at the central point was used to initially assess the impact of extraction parameters as follows: (1) the ratio of plant mass to NADES, (2) the extraction temperature, and (3) the extraction time. The response used to determine the impact of the parameters was the total peak of the chromatograms and was evaluated with 95% significance (Table 3).

**Figure 3:**
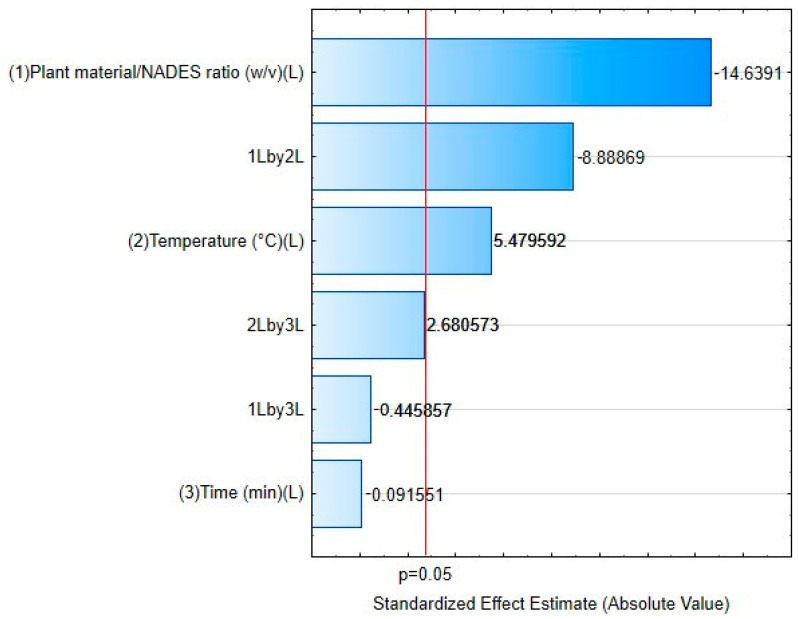
Pareto chart of the effects obtained as a result of the analysis of the 2^3^ full factorial design.

**Figure 4:**
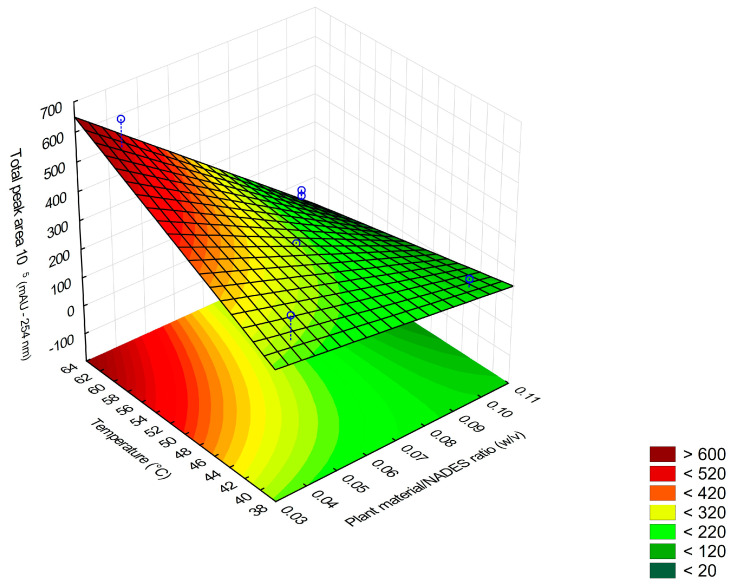
Surface graph obtained from 2^3^ full factorial design for ratio plant material/NADES ratio (X_1_) and temperature (X_2_) variables.

From the responses obtained, a Pareto chart was plotted to evaluate the results of the effects between the studied variables (Figure 3). At the 95% confidence level, the variables plant/NADES ratio (X_1_) and temperature (X_2_) significantly affected the response and should be at their lowest (−) and highest (+) levels, respectively; the time relationship (X_3_) did not significantly affect the response. With this, an RSM was obtained as a function of the variables X_1_ and X_2_ (Figure 4).

From the RSM, it was possible to create a mathematical model based on variables X_1_ and X_2_ from the fitting of the measured values (Equation (1)):

A = 2.63 × 10^7^ − 1.41 × 10^7^X_1_ + 5.29 × 10^6^X_2_ − 8.59.10^7^X_1_X_2_ (1)

where A is the sum of the total area of the peaks, X_1_ is the plant material/NADES ratio (*w*/*v*), and X_2_ is the temperature (°C).

ANOVA, calculated by Excel 2013 software, is an essential statistical tool for evaluating whether the calculated model is appropriate for describing experimentally observed data. This analysis compares the variation in responses with random errors, allowing the significance of the relationships between the variables in the model to be determined [34]. For this dataset, the objective was to verify whether the linear regression model is adequate to describe the data based on the regression and residuals (Table 4). After the Fisher Distribution test at a 95% confidence level, the results revealed that the calculated F value (8.40) was greater than the tabulated F value (4.88), indicating a poor fit. Furthermore, it was observed that the calculated F (24.98) was greater than the tabulated F (7.71), which further confirms a lack of fit [34]. This lack of fit indicates that the linear model does not accurately describe the experiments. Such inadequacy is demonstrated by the curvature test, which indicates that there is a curvature or non-linearity in the data that is not captured by the linear regression model. This suggests that a more complex model, which considers the curvature present in the data, should be adopted for a more accurate description of the phenomenon under study.

The RSM methodology is divided into two steps, which can be repeated until the optimal region of the investigated surface is reached. These steps are as follows: (1) modeling, which involves the adjustment of simple models, and (2) displacement, in which the exploration is done in the direction of the maximum inclination of the model [34]. Based on the response surface obtained and the ANOVA, it became relevant to carry out the displacement in the direction of maximum inclination (in essence, where the plant/NADES ratio is lowest and the temperature is highest). The purpose of the displacement was to optimize the extraction, allowing for the identification of the optimal region of the investigated surface. Through these controlled displacements, it was possible to further demonstrate how the variables plant/NADES ratio and temperature affect extraction yield.

From the model, b2/b1 was calculated, which resulted in −1.47.107/5.29.106 = −2.671. This means that, for each unit change in variable X_1_, variable X_2_ must increase by 2.671 units. These displacements were applied, resulting in the experiments presented in Table 5.

Based on the results obtained in the displacement and the Pareto chart (Figure 4), a new complete factorial planning was carried out, later transforming it into a CCD with the addition of axial points, utilizing a quadratic model. Since variable X_3_ (the extraction time) had no significant impact on the previous model, the CCD was only used to study the impact of the plant/NADES ratio (X_1_) and the extraction temperature (X_2_). A maximum of 70 °C was used for the optimization, due to the equipment limitations, which is described as the α level in the CCD.

**Figure 5:**
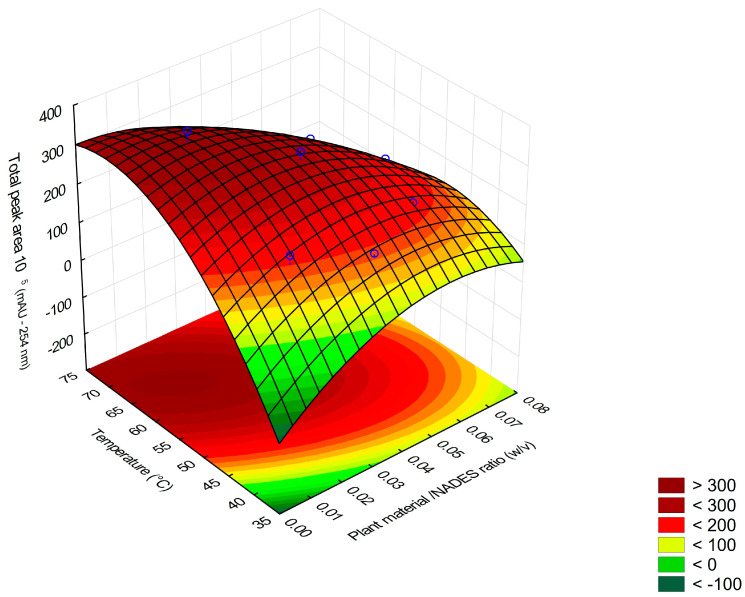
Response surface obtained for the CCD.

By initially screening for significant variables and then focusing on only two variables, the complexity of the development was reduced, with the additional green advantage of saving resources. The inclusion of axial points in the planning, which resulted in the CCD (Table 6), made it possible to obtain additional information about the curvature of the RSM (Figure 5), as the quadratic model can capture non-linearities in the relationships between the variables.

With the additional values added to the model, it was possible to calculate a new mathematical model (Equation (2)):

A = 2.89 × 10^7^ + 2.80 × 10^6^X_1_ − 2.65 × 10^6^X_2_ − 3.60 × 10^6^X_12_ − 3.04 × 10^6^ X_1_^2^ − 2.21 × 10^6^X_2_^2^ (2)

where A is the sum of the total area of the peaks, X_1_ is the plant material/NADES ratio (*w*/*v*), and X_2_ is the temperature (°C).

When performing the ANOVA for the new model obtained by the CCD (Table 7), the quadratic means related to the regression and residuals and the Fisher Distribution test were calculated again at a confidence level of 95%. The calculated F value (12.09) was greater than the tabulated F value (5.05), indicating significance for the regression. This means that the new regression model used has a statistically significant relationship with the observed data. Furthermore, the calculated F value (1.85) is lower than the tabulated F value (19.16), indicating no significance for the lack of adjustment. In this case, we can infer that the model adequately fits the experimental data, with no statistical evidence of significant lack of fit.

Additionally, the curvature test was performed again and indicated the existence of non-linearities in the data. Together with the information obtained by ANOVA, it was concluded that the quadratic model is more appropriate to represent the behavior of the system studied, providing a more accurate understanding of the system and, consequently, facilitating the search for the ideal extraction conditions. Therefore, based on the analyses carried out, the quadratic model is considered adequate to represent the relationship between the variables X_1_ (plant material/NADES ratio) and X_2_ (temperature) and the extraction yield. This information makes it possible to optimize variables in a green design, allowing the best conditions for the extraction process to be obtained.

Based on RSM using the quadratic model and the desirability mode in the Statistica 10 software, the best extraction condition by MAE was determined, with the most favorable combination of parameters to obtain the best extraction result, using the method: X_1_ = 0.02 (i.e., a proportion of 1:50 *w*/*v*); X_2_ = 70 °C; X_3_ = 15 min.

### 2.4. Comparison of Efficiency and Environmental Impact between the Developed Method and Reference Methods

**Figure 6:**
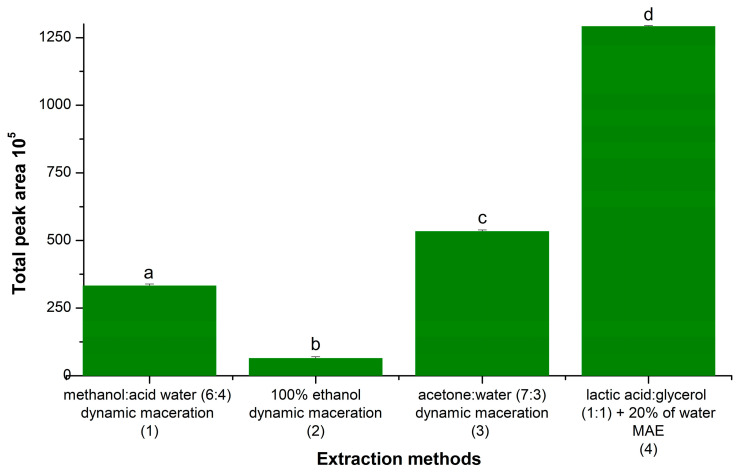
Comparison between NADES–MAE extraction method and reference extraction methods. Different letters represent a statistically significant difference: a *p* < 0.0005 (1) dynamic maceration with methanol/acid water [24]; (2) dynamic maceration with ethanol [36]; (3) dynamic maceration with acetone/water [35]; (4) MAE with NADES (lactic acid/glycerol/water).

Established literature methods were compared with the developed method using MAE and the NADES LA:GLY (N9). In terms of efficiency, the developed method extracted a greater quantity of target compounds than the reference methods, shown by the increase in total peak area in the chromatograms (Figure 6, Table S2). Of the reference methods, the method by Meshikini [35] (acetone and water extraction solvent) was the one that presented the greatest extraction efficiency; however, the NADES–MAE method in this work had an approximately 2.4 times greater extraction yield, indicating that the NADES–MAE combination has greater analytical efficiency for this application. Moreover, using this NADES–MAE approach can represent a significant advance in the extraction of phenolic compounds in plant matrices compared to traditional methods in the literature.

**Figure 7:**
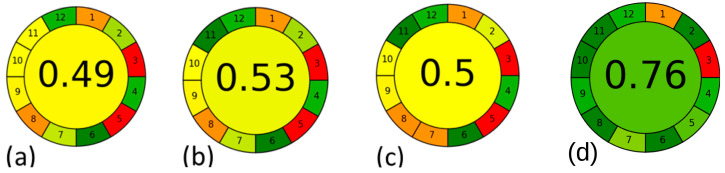
Pictograms with the scores obtained by the AGREE metric v.0.4.2020 of the extractor methods: (**a**) dynamic maceration with methanol/acid water [24]; (**b**) dynamic maceration with ethanol [36]; (**c**) dynamic maceration with acetone/water [35]; (**d**) MAE with NADES (lactic acid/glycerol/water).

Regarding environmental impact, the NADES–MAE method was the one that obtained the highest score (0.75) from the AGREE metric (Figure 7, Table S3), indicating that it was the greenest method among the compared methods. The higher scores for the NADES–MAE method was due to the use of low toxicity solvents, high extraction efficiency, and low energy consumption (from the MAE technique). Only two penalties were obtained regarding principles 1 and 3, which are related to the following: (1) sample treatment, in which pre-treatment occurs for subsequent analysis, where the ideal situation would be to have no sample pre-treatment; (3) types of measurements, in which the sample was analyzed in the off-line mode (manual sample preparation and automated extraction), while the most desirable way would be an in-line measurement (sample preparation and extraction occur together and in a completely automated way).

**Figure 8:**
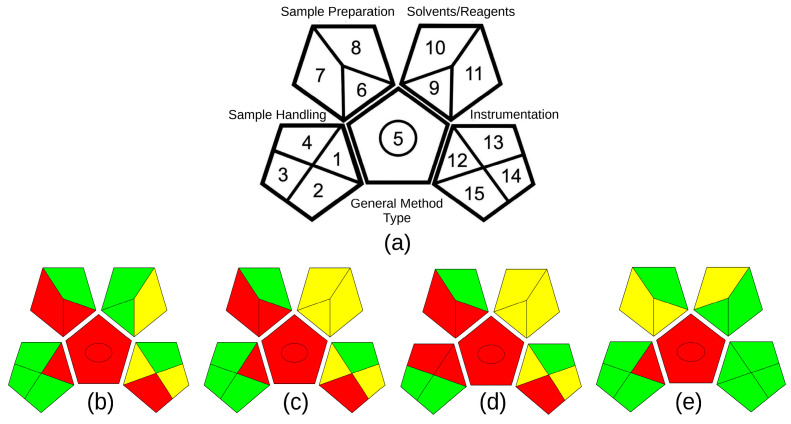
Pictograms obtained by the ComplexGAPI v.0.2 beta of the extractor methods: (**a**) reference pictogram; (**b**) dynamic maceration with methanol/acid water [24]; (**c**) dynamic maceration with ethanol [36]; (**d**) dynamic maceration with acetone/water [35]; (**e**) MAE with NADES (lactic acid/glycerol/water).

When analyzing the results obtained by the GAPI metric, it was found that the method developed also presented a greater green character, due to the presence of a greater number of fields filled in green in the pictogram (Figure 8, Table S4). Again, the penalties obtained in this metric are related to the need to collect study material and prepare the sample for subsequent analysis, while in situ measurements would be desirable.

## 3. Materials and Methods

### 3.1. Chemicals

Extraction: acetone (AR grade, Synth^®^, Diadema, SP, Brazil), ethanol (EtOH), and methanol (MeOH), all HPLC grade (LiChrosolv, MerckKGaA, Darmstadt, Germany), ultra-purified water from a Milli-Q system (Millipore, Burlington, MA, USA), glycerol (GLY) ≥ 99.5%, d-glucose (GLU) ≥ 99.5%, sucrose (SAC) ≥ 99.5%, lactic acid (LA) ≥ 85%, d-sorbitol (SOR) ≥ 98%, d-malic acid (MA) ≥ 98%, choline chloride (CL) ≥ 98%, d-fructose (FRU) ≥ 99%, l-proline (PRO) ≥ 99%, l-menthol (MEN) ≥ 99%, thymol (THY) ≥ 99% (all Sigma Aldrich, St. Louis, MO, USA).

Mobile phase HPLC: ethanol (EtOH, HPLC grade) (LiChrosolv, MerckKGaA, Darmstadt, Germany), ultra-purified water from a Milli-Q system and acetic acid (AcOH, HPLC grade) (LiChrosolv, MerckKGaA, Darmstadt, Germany).

### 3.2. Plant Material

Whole almond nuts including their skins, shells, and hulls were obtained from the producers Yunis Pty (Salisbury North, SA, Australia), CMV farms (Adelaide, SA, Australia), and Taronga Almonds (McLaren Vale, SA, Australia) from summer and autumn harvests in 2019–2021. Almonds were stored at 4 °C in closed containers prior to having the hull and shells separated.

### 3.3. Sample Preparation

The hulls were separated, cleaned, placed in equal portions, and frozen at a temperature of −20 °C. They were subsequently freeze-dried in a Christ freeze dryer (model Alpha 12LDplus Gefriertrocknungsanlagen, Osterode am Harz, Germany) for a period of 7 days. After lyophilization, the resulting material was pulverized in a knife mill and separated into a powder with particle sizes between 250 and 850 μm.

### 3.4. Reference Extraction Methods

Three reference methods of extracting secondary metabolites from almond by-products that have multiple citations in the literature were reproduced, with adaptations. Firstly, the method from Pinelo et al. [24] using methanol and water with an acidic pH as an extracting solvent was used. Secondly, the method from Rubilar et al. [36] using ethanol as the extraction solvent was used. Lastly, the method from Meshkini [35] using acetone as the extraction solvent was reproduced. In all methods, dynamic maceration was applied as the extraction technique. Extractions were adapted to microscale and performed using a temperature-controlled Heidolph^®^ magnetic stirrer (Schwabach, Baviera, Germany) (using 0.1 g of powder, following the solvent volume, temperature, and rpm from each reference).

After dynamic maceration, the extracts were centrifuged at 7200 rpm in a mini-centrifuge (BioPet^®^, Knoxville, TN, USA), and approximately 1 mL of supernatant was collected and filtered through a 0.45 µm PTFE microfilter (Phenomenex^®^, Torrance, CA, USA) into 2 mL vials for further analysis by High-Performance Liquid Chromatography (HPLC) coupled to a PhotoDiode Array Detector (HPLC–DAD).

### 3.5. High-Performance Liquid Chromatography (HPLC–DAD) System

HPLC–DAD analyses were carried out on a Shimadzu system (LC-20AT pump, DGU-20A5R degasser, SIL-20HT sampler, CTA-10AS VP column oven, and interface CBM-20A, Shimadzu, Kyoto, Japan), equipped with a Luna column (2) C18-Phenomenex (250 × 4.6 mm), and a photodiode array detector (DAD, SPD-M20A), using 1% *v*/*v* AcOH in water and EtOH as the mobile phases, A and B, respectively. The samples were analyzed in gradient mode, with B varying from 5% to 60% in 60 min at a flow rate of 1 mL∙min^−1^. The oven temperature was 30 °C. A 50 µL injection volume was used. The detection wavelength was 254 nm. The equilibration was achieved under the initial conditions of the gradient, with 5% B, a flow rate of 1 mL∙min^−1^, and the column at 30 °C, for 15 min. The wash out was 10 min, with 100% B passing through the column.

### 3.6. Identification of Compounds Present in the Hydroethanolic Extract of Almond Hulls

The identity of the peaks in the chromatogram was confirmed from UV spectra obtained using the HPLC–DAD system and by liquid chromatography coupled to high-resolution mass spectrometry (LC–HRMS).

LC–HRMS analyses were performed using the conditions described in Section 3.5 using an Agilent 1200 LC–6520 QTOF HPLC system with an electrospray ionization (ESI) (Agilent Technologies, Santa Clara, CA, USA), source in negative mode. A capillary voltage of 3.5 kV and N_2_ gas flow rate (10 L∙min^−1^) at 280 °C was used. The nebulizer was set to 45 psi; the fragmentor voltage was 155 V. N_2_ was used as the collision gas (30 eV). MassHunter B.07 software (Agilent^®^) was used to acquire and process the data acquired.

### 3.7. Preparation of Natural Deep Eutectic Solvents (NADESs)

The preparation of NADESs was based on the methodology of Gomez et al. [37] via microwaves, which consists of subjecting the mixture to microwave radiation, varying and optimizing parameters such as irradiation time, temperature, and power. Two or three starting components at specific molar ratios and with water (Table 8) were placed in borosilicate vials (4 cm i.d., 5 mL), then introduced into Teflon tubes (24.5 cm × 5 cm) prior to being subjected to microwave irradiation (Ethos Easy, Milestone SrL, Milan, Italy) at low power (200 W) in 5 min cycles (3 min of ramp to reach the programmed temperature of 40 or 50 °C followed by 2 min of irradiation with a stable temperature). The cycles were repeated for each NADES until a homogeneous and transparent solution was obtained. The mass percentage of water in the eutectic solvents was 20% (*w*/*w*), as adding water in quantities exceeding this amount can result in the disruption of hydrogen bonds present. This, in turn, leads to the dissolution of the supramolecular structure of the NADES, as observed in previous studies [30,31]. The resulting solutions were used as extraction solvents.

### 3.8. Conditions Used in NADES Screening

To identify the NADESs with the greatest extractive capacity, all studied NADESs (Table 8) were used to extract secondary metabolites present in almond by-products, using microwave irradiation in triplicates. For every 0.1 g of almond hull powder, 1 mL of NADES extraction solution was added in a borosilicate vial (4 cm i.d., 5 mL) and introduced into Teflon tubes (24.5 cm × 5 cm). All extractions were carried out under microwave irradiation for 60 min as follows: 10 min of ramp to reach the programmed temperature, followed by 50 min of irradiation with a stable temperature at 50 °C and 500 W. Due to the high viscosity of most NADESs, after extraction, the mixtures were diluted in EtOH:H_2_O (7:1, *v*:*v*) so that they all had the same final concentration, placed in 1.5 mL microtubes (Eppendorf, Hamburg, Germany, Olen, EUA), and centrifuged in a microcentrifuge (Biopet Technologies, Brazil) for 3 min at 7200 rpm. This process was carried out twice to obtain a more homogeneous supernatant. After centrifugation, 500 µL of supernatant was collected, filtered through a 0.45 µm PTFE microfilter (Phenomenex, USA), and analyzed with the HPLC–DAD method described in Section 3.5.

### 3.9. Experimental Design

A complete 2^3^ factorial design with repetition at the central point was carried out to evaluate the significant variables and perform a curvature test for subsequent optimization. The independent variables studied are presented in Table 2.

The response used in this study was the sum of the peak areas corresponding to the target compounds (tannins and catechin monomers) obtained from the HPLC–DAD analysis with detection at λ = 254 nm. Furthermore, the response (total peak area of the chromatogram) was evaluated at a 95% significance level. A central composite design (CCD), using the significant variables, was performed to globally optimize the extraction conditions. At this stage, the power was set at 500 W, according to the power used in the NADES screening (Section 3.8).

Analysis of Variance (ANOVA) calculations, and Response Surface plots (RSM) were performed using Statistica 10 software (Stat-Ease, Inc., Minneapolis, MN, USA), Origin Pro version 8.5, and Excel 2013 (Microsoft, Washington, DC, USA).

### 3.10. Assessment of Method Greenness

The comparison between the method developed in this work and the reference methods was carried out using two green metrics: Analytical GREEness (AGREE) (v.0.4.2020, Vigo, Pontevedra, Spain) and Green Analytical Procedure Index (GAPI) ComplexGAPI (v.0.2 beta Gdańsk, Poland).

The AGREE metric uses pictograms with colors that vary from green to red and shades that are calculated on a scale of 0 to 1, 1 being a highly green method (represented by dark green coloring) and 0 representing a lack of green chemistry principles (represented by red color) [38].

The GAPI metric also uses pictograms with a color scale to classify the degree of greenness of each step of an analytical procedure, with three levels of evaluation for each step. GAPI has five pentagrams to evaluate and quantify the environmental impact as low, medium, and high at each stage of the methodology, using the colors green, yellow, and red, respectively. Each field reflects a different aspect of the described analytical procedure and is filled in green if certain requirements are met [39,40].

## 4. Conclusions

It is concluded that the method developed in this work was created based on a multivariate optimization which is a greener method design than a univariate optimization. The method employs an easy-to-prepare NADES (lactic acid/glycerol 1:1 with 20% water) as the extraction medium and MAE as the technique. The NADES–MAE method was more efficient and greener for the extraction of catechin, caffeoylquinic acid, and condensed tannins from almond by-products when compared to reference methods. Furthermore, these by-products have the potential to become long-term sources of the metabolites extracted, adding value to the almond production chain by means of this green extraction method.

## Figures and Tables

**Table 1:** Compounds referring to the observed ions.

*m*/*z*	Compounds
289.0715	Catechin/epicatechin monomer
577.1346	Catechin/epicatechin dimer
865.1979	Catechin/epicatechin trimer
1153.2609	Catechin/epicatechin tetramer
1441.3240	Catechin/epicatechin pentamer
353.0872	Caffeoylquinic acid

**Table 2:** Full factorial design variables 2^3^.

	Variables		Levels	
		−1	0	1
X_1_	Plant material/NADES ratio (*w*/*v*)	0.04	0.07	0.1
X_2_	Temperature (°C)	40	51	62
X_3_	Time (min)	15	40	65

**Table 3:** Coded (real in parenthesis) values for the variables studied in the 2^3^ full factorial design and respective response.

Experiment	Independent Variables			Response
	X_1_ ^a^	X_2_ ^a^	X_3_ ^a^	Total Area ± RSD ^b^
1	−1 (0.04)	−1 (40 °C)	−1 (15 min)	348.85 ± 2.74
2	+1 (0.1)	−1 (40 °C)	−1 (15 min)	184.79 ± 3.32
3	−1 (0.04)	+1 (62 °C)	−1 (15 min)	513.31 ± 9.58
4	+1 (0.1)	+1 (62 °C)	−1 (15 min)	128.56 ± 1.50
5	−1 (0.04)	−1 (40 °C)	+1 (65 min)	242.37 ± 1.06
6	+1 (0.1)	−1 (40 °C)	+1 (65 min)	184.08 ± 4.98
7	−1 (0.04)	+1 (62 °C)	+1 (65 min)	633.49 ± 2.17
8	+1 (0.1)	+1 (62 °C)	+1 (65 min)	108.49 ± 0.28
9 ^c^	0 (0.07)	0 (51 °C)	0 (40 min)	258.34 ± 1.01
10 ^c^	0 (0.07)	0 (51 °C)	0 (40 min)	194.59 ± 2.39
11 ^c^	0 (0.07)	0 (51 °C)	0 (40 min)	220.47 ± 0.96
12 ^c^	0 (0.07)	0 (51 °C)	0 (40 min)	212.69 ± 1.11
13 ^c^	0 (0.07)	0 (51 °C)	0 (40 min)	189.29 ± 4.68

^a^ X_1_ (plant material/NADES ratio, *w*/*v*); X_2_ (temperature, °C); X_3_ (extraction time with a 5 min ramp for all, min); ^b^ RSD is the relative standard deviation referring to the average (×10^5^) of three HPLC––DAD analyses with 50 µL injection volume; ^c^ central points.

**Table 4:** ANOVA for the estimated model.

				Test F		
	Quadratic Sum	Degrees of Freedom	Means Square	Calculated	Tabulated	*p*-Value
Regression	2.54 × 10^15^	7	3.64 × 10^14^	8.40	4.88	0.0162
Residue	2.17 × 10^14^	5	4.33 × 10^13^			
Lack of fit	1.87 × 10^15^	1	1.87 × 10^14^	24.98	7.71	0.0075
Pure error	2.99 × 10^13^	4	7.48 × 10^12^			
Total	2.76 × 10^15^	12				
R^2^	0.87					
Curvature Check						
	Average	Ce ^a^	Variance	s ^b^	(+)	(−)
Factorial	2.93 × 10^7^	7.79 × 10^6^	2.43 × 10^12^	1.56 × 10^6^	9.35 × 10^6^	6.23 × 10^6^
Central point	2.15 × 10^7^					

^a^ Ce: curvature estimative; ^b^ s: standard deviation.

**Table 5:** Maximum inclination for the calculated model (Equation (1)).

	Coded Value		Real Value		
Experiment	X_1_ ^a^	X_2_ ^a^	X_1_ ^a^	X_2_ ^a^	Total Area ± RSD ^b^
1	0	0	0.07	51.0	197.64 ± 2.31
2	−1	2.671	0.06	60.6	266.65 ± 2.45
3	−2	5.342	0.05	70.2	384.61 ± 3.22
4	−3	8.013	0.04	79.8	343.91 ± 1.89
5	−5	13.355	0.02	99.1	262.13 ± 4.29

^a^ X_1_: plant material/NADES ratio (*w*/*v*), X_2_: temperature (°C); ^b^ RSD is the relative standard deviation referring to the average (×10^5^) of three HPLC–DAD analyses with 50 µL injection volume.

**Table 6:** Central composite design (CCD).

Experiment	Independent Variables		Response
	X_1_ ^a^	X_2_ ^a^ (°C)	Total Area ± RSD ^b^
1	−1 (0.02)	−1 (45)	200.21 ± 1.26
2	+1 (0.06)	−1 (45)	193.83 ± 3.52
3	−1 (0.02)	+1 (65)	348.66 ± 2.33
4	+1 (0.06)	+1 (65)	198.07 ± 2.46
CP ^c^	0 (0.04)	0 (55)	277.01 ± 5.69
CP ^c^	0 (0.04)	0 (55)	281.04 ± 4.11
CP ^c^	0 (0.04)	0 (55)	310.13 ± 2.54
5	−α (0.01)	0 (55)	256.82 ± 2.67
6	+α (0.07)	0 (55)	197.89 ± 1.52
7	0 (0.04)	−α (41)	168.80 ± 3.21
8	0 (0.04)	+α (69)	238.92 ± 1.47

^a^ X_1_: ratio of plant material/NADES (*w*/*v*), X_2_: temperature (°C); ^b^ RSD is the relative standard deviation referring to the average (×10^5^) of three HPLC–DAD analyses with 50 µL injection volume; ^c^ central points.

**Table 7:** ANOVA for the CCD.

				Test F		
	Quadratic Sum	Degrees of Freedom	Means Square	Calculated	Tabulated	*p*-Value
Regression	2.98 × 10^14^	5	5.96 × 10^13^	12.09	5.05	0.008
Residue	2.46 × 10^13^	5	4.93 × 10^12^			
Lack of fit	1.81 × 10^14^	3	6.04 × 10^12^	1.85	19.16	0.3696
Pure error	6.53 × 10^13^	2	3.26 × 10^12^			
Total	2.23 × 10^14^	10				
R^2^	0.92					
Curvature Check						
	**Average**	**Ce ^a^**	**Variance**	**s ^b^**	**(+)**	**(−)**
Factorial	2.25 × 10^7^	8.48 × 10^6^	1.49 × 10^12^	1.22 × 10^6^	−5.17 × 10^6^	−7.62 × 10^6^
Central point	2.89 × 10^7^					

^a^ Ce: curvature estimative; ^b^ s: standard deviation.

**Table 8:** Composition of natural deep eutectic solvents (NADESs) prepared.

NADES	Component 1 ^a^	Component 2 ^a^	Component 3 ^a^	Molar Ratio	H_2_O (%)	Coloration
N1	MA	SOR		1:1	20	Colorless
N2	MA	GLU	FRU	1:1:1	20	Slightly yellowish
N3	LA	SUC		2:1	20	Slightly yellowish
N4 ^b^	LA	GLU		2:1	20	Colorless
N5	CL	SOR		1:2	20	Colorless
N6	CL	GLU		1:1	20	Colorless
N7	PRO	MA		1:1	20	Slightly yellowish
N8	GLY	CL		2:1	20	Colorless
N9	LA	GLY		1:1	20	Colorless
N10	THY	MEN		1:1	-	Slightly yellowish

^a^ CL: choline chloride, FRU: fructose, GLU: glucose, GLY: glycerol, LA: lactic acid, MA: malic acid, MEN: menthol, PRO: proline, SOR: sobitol, THY: thymol; ^b^ N4 did not produce a stable liquid, as after 7 days it became solid—therefore, N4 was not evaluated in subsequent studies.

## Data Availability

The data presented in this study are available in article.

## Supplementary Materials

The following supporting information can be downloaded at: https://www.mdpi.com/article/10.3390/molecules29133034/s1. Figure S1: LC-HRMS chromatograms of the almond hull of the extract: (a) total ion current; (b) monitored at *m*/*z* 289; (c) monitored at *m*/*z* 577; (d) monitored at *m*/*z* 865 and (e) monitored at *m*/*z* 1153 (HPLC-DAD conditions, 3.6); Figure S2: LC-HRMS chromatograms of the almond hull of the extract: (a) total ion current; (b) monitored at *m*/*z* 353 (HPLC-DAD conditions, 3.6): Figure S3: LC-HRMS fingerprint spectra obtained in negative ion mode of hlmond hull extract corresponding to procyanidins; Figure S4: LC-HRMS fingerprint spectra obtained in negative ion mode of hlmond hull extract corresponding to caffeioquinic acid; Table S1: Data from the areas related to the graph in the Figure 2; Table S2: Data from the areas related to the graph in the Figure 6; Table S3: Input parameters in the AGREE metric software to elucidate the pictograms referring to Figure 7; Table S4: Input parameters in the GAPI metric software to elucidate the pictograms referring to Figure 8.
