# Supplementary material for: Sustainable and Biomimetic Methodology for Extraction of High-Value-Added Compounds in Almond Hulls
Source: Molecules. 2024 Jun 26;29(13):3034. doi: 10.3390/molecules29133034 (PMC11243185; doi:10.3390/molecules29133034)
Supplement: Supplementary file 1 [file molecules-29-03034-s001.zip › molecules-2995949-supplementary.pdf]

molecules

MDPI

Type of the Paper (Article, Review, Communication, etc.)

Sustainable and biomimetic methodology for extraction of high value-added compounds in almond hulls

Gabriela Cremasco[1], Adam T. Sutton[2], Cristiano S. Funari[3], Dario Arrua[2], Kelly J. Dussan[1], Emily F. Hilder[2], Vanderlan S. Bolzani[1], and Daniel Rinaldo[1,4*]

[1] São Paulo State University (UNESP), Institute of Chemistry, R. Prof. Francisco Degni 55, Araraquara, SP, 14800-900, Brazil
[2] University of South Australia (UniSA), Future Industries Institute, X Building, Mawson Lakes, SA, 5095, Australia
[3] University of South Australia (UniSA), Future Industries Institute, X Building, Mawson Lakes, SA, 5095, Australia
[4] Green Biotech Network, São Paulo State University (UNESP), School of Sciences, Av. Eng. Luiz Edmundo Carrijo Coube 14-01, Bauru, SP, 17033-360, Brazil
* daniel.rinaldo@unesp.br

Supplementary Materials:

Figure S1: LC-HRMS chromatograms of the almond hull of the extract: a) total ion current; b) monitored at m/z 289; c) monitored at m/z 577; d) monitored at m/z 865 and e) monitored at m/z 1153 (HPLC-DAD conditions, 3.6).

a)

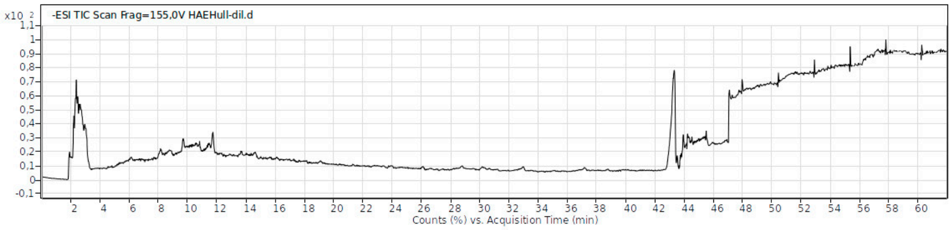

b)

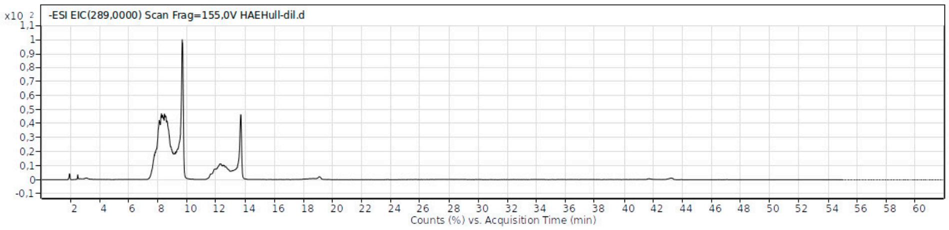

c)

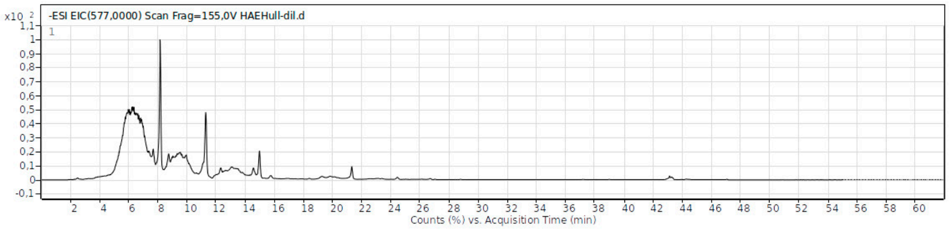

d)

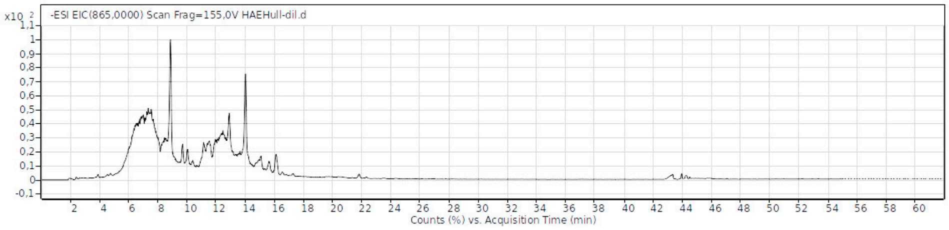

e)

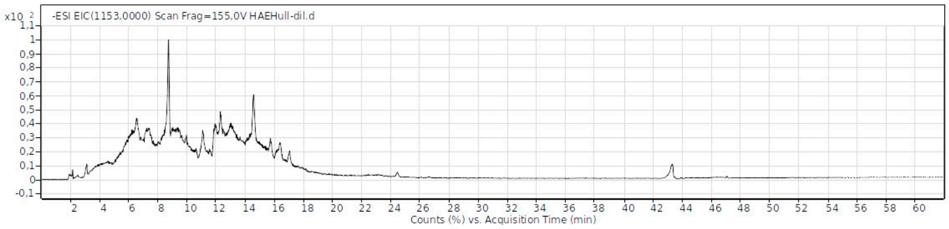

Figure S2: LC-HRMS chromatograms of the almond hull of the extract: a) total ion current; b) monitored at m/z 353 (HPLC-DAD conditions, 3.6).

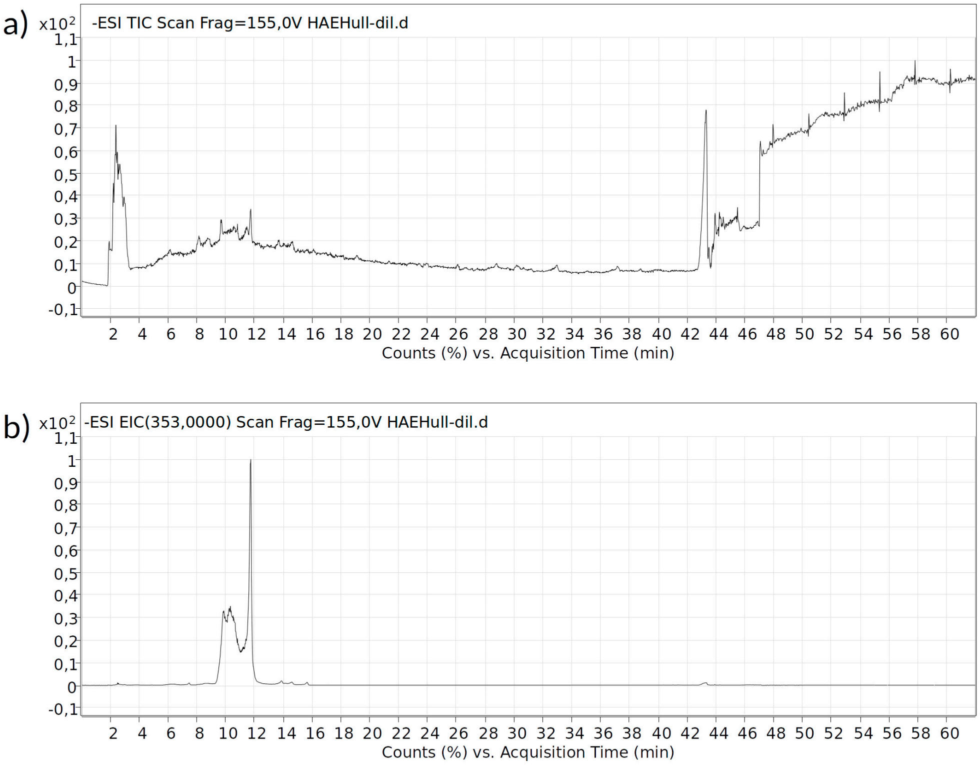

Figure S3: LC-HRMS fingerprint spectra obtained in negative ion mode of hlmond hull extract corresponding to procyanidins.

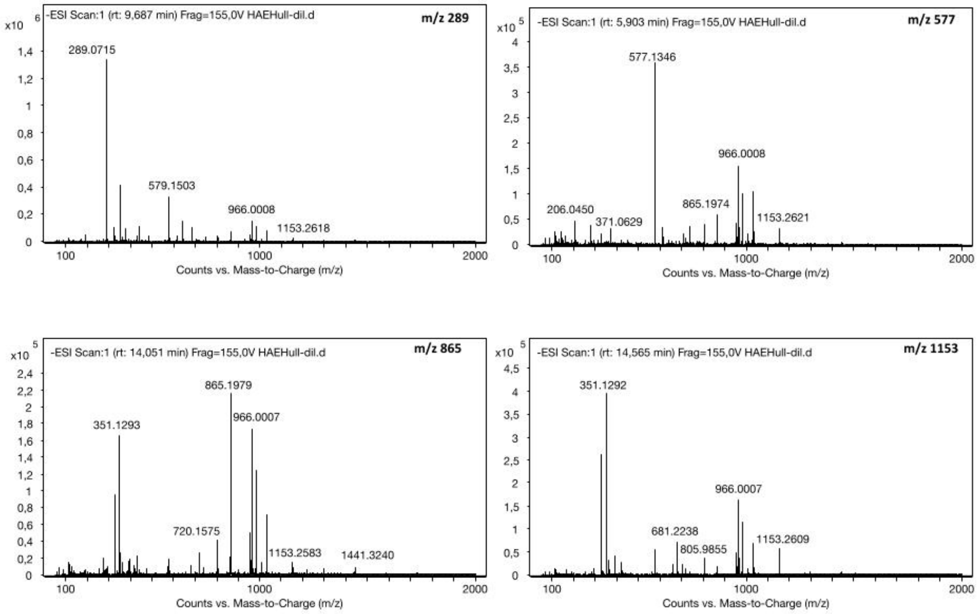

Figure S4: LC-HRMS fingerprint spectra obtained in negative ion mode of hlmond hull extract corresponding to caffeioquinic acid.

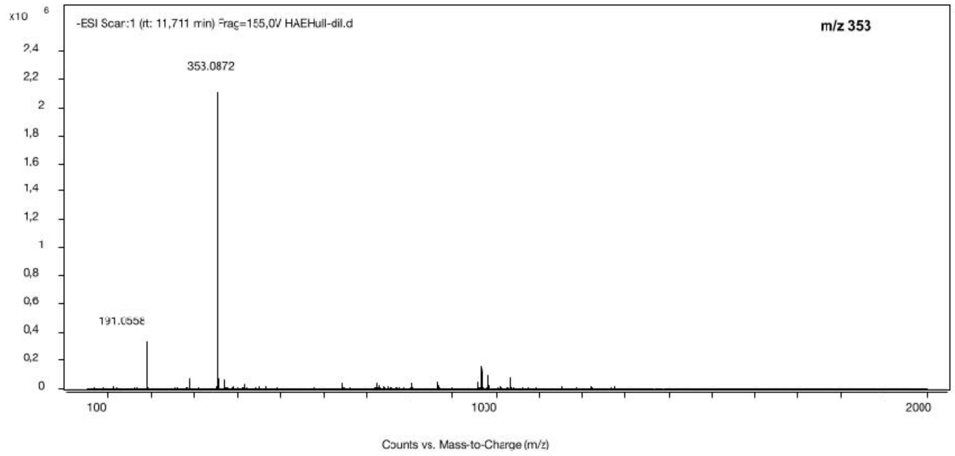

Table S1: Data from the areas related to the graph in the Figure 2.

NADES	Total area* (.10^5)	Standard deviation
**MA:SOR (1:1)**	190,94	1,37
**MA:SOR:GLU (1:1:1)**	308,76	2,56
**LA:SUC (2:1)**	474,12	1,61
**CL:SOR (1:2)**	227,08	8,86
**CL:GLU (1:1)**	376,19	2,22
**PRO:MA (1:1)**	383,57	6,82
**GLY:CL (2:1)**	520,35	4,38
**LA:GLY (1:1)**	666,37	6,67
**THY:MEN**	0,00	-

* Average relative to triplicate

Table S2: Data from the areas related to the graph in the Figure 10.

	Total area* (.10^5)	Standard deviation
**Methanol:acid water (6:4) / dynamic maceration**	332,22	6,51
**100% etanol / dynamic maceration**	64,33	5,88
**Acetone:water (7:3) / dynamic maceration**	533,79	4,91
**Lactic acid:glycerol (1:1) + 20% of water / MAE**	1291,70	2,17

* Average relative to triplicate

Table S3: Input parameters in the AGREE metric software to elucidate the pictograms referring to Figure 7.

Princípios GAC	(a)	(b)	(c)	(d)	(e)
**1 – sample treatment**	0,3	0,3	0,3	0,3	0,3
**2 – sample size**	0,65	0,65	0,55	0,65	0,98
**3 - *in situ* measurements**	0,0	0,0	0,0	0,0	0,33
**4 – Analytical processes**	0,8	0,8	0,8	0,8	0,8
**5 - automated and miniaturized methods**	0,0	0,0	0,0	0,0	0,75
**6 - derivatization**	1,0	1,0	1,0	1,0	1,0
**7 – analyticas management**	0,6	0,6	0,29	0,6	0,6
**8 – multianalyte methods**	0,29	0,29	0,29	0,29	1,0
**9 – use of energy**	0,48	0,48	0,48	0,48	0,8
**10 – used reagentes**	0,5	0,5	0,5	1,0	1,0
**11 – toxic reagents**	0,5	1,0	1,0	1,0	1,0
**12 – safety os the operator**	0,8	0,8	0,8	0,8	0,8

Table S4: Input parameters in the GAPI metric software to elucidate the pictograms referring to Figure 8.

	(b)	(c)	(d)	(e)	(f)
**Collection**	off-line	off-line	off-line	off-line	off-line
**Preservation**	None	None	None	None	None
**Transport**	None	None	None	None	None
**Storage**	None	None	Under special conditions	Under special conditions	None
**Type of method**	Extraction	Extraction	Extraction	Extraction	Extraction

**Scale**	Macro	Macro	Macro	Macro	Micro
**Solvents**	Non-green	Non-green	Non-green	Non-green	Green
**Additional**	None	None	None	None	None
**Amount**	<10 mL/g	10 - 100 mL/g	10 - 100 mL/g	10 - 100 mL/g	<10 mL/g
**Health**	NFPA = 0 ou 1	NFPA = 2 ou 3	NFPA = 0 ou 1	NFPA = 2 ou 3	NFPA = 2 ou 3
**Safety**	NFPA = 2 ou 3	NFPA = 2 ou 3	NFPA = 2 ou 3	NFPA = 2 ou 3	NFPA = 1 ou 2
**Energy**	≤1.5 Kw for sample	≤1.5 Kw for sample	>1.5 Kw for sample	≤1.5 Kw for sample	≤0.1 Kw for sample
**Occupational**	Hermetic sealing	Hermetic sealing	Hermetic sealing	Hermetic sealing	Hermetic sealing
**Waste**	1 - 10 mL/g	1 - 10 mL/g	1 - 10 mL/g	1 - 10 mL/g	<1 mL/g
**Waste treatment**	None	None	None	None	Recycling
